# MacroLoops for microRNAs: shall we revise the maximum allowed size?

**DOI:** 10.1093/plcell/koae017

**Published:** 2024-01-22

**Authors:** Laura Arribas-Hernández

**Affiliations:** Assistant Features Editor, The Plant Cell, American Society of Plant Biologists; Biology Department, University of Copenhagen, Ole Maaløes Vej 5, 2200 Copenhagen N, Denmark

When it comes to RNA, size matters. So much so, that the shortest among these molecules are named just after their size—we simply call them *microRNAs* (miRNAs). But do not let their stature deceive you. miRNAs can destroy much larger RNAs once they find their Achilles' heel: an exposed stretch of nucleotides with enough complementarity to their own sequences. By base-pairing to these target sites, miRNAs bring in endoribonucleolytic enzymes to slice their victims. As treacherous as this may sound, miRNA activity is indispensable for ridding cells of messenger RNAs no longer needed, and this function is essential for the correct development of plants, animals, and other organisms.

Due to their importance, the scientific community has spared no effort in identifying miRNAs and their precursors. The task is far from trivial: a mature miRNA molecule is one of the two ∼21-nucleotide-long strands of an imperfectly complementary miRNA duplex that, in turn, has been excised from the stem of a hairpin structure from a longer precursor. Until now, the size of such stem-loops in plants was thought to be quite modest, shorter than 336 nucleotides for the vast majority. Consequently, standard bioinformatics tools used for the identification of miRNA precursors do not consider long-looped hairpins as viable candidates. In this issue, **Wen-qiu Wang and colleagues** (Wang et al. 2024) challenge this view, as they report the widespread occurrence among seed plants of non-canonical hairpins with long loops that give rise to miR858.

miR858 is important. It regulates the metabolism of polyphenolic compounds such as lignin—the essential constituent of wood and bark—and the flavonols that give color to flowers and fruits (Xia et al. 2012, Sharma et al. 2016). While studying miR858 precursors in kiwi fruit, Wang et al. (2024) realized that none of the four genomic locations containing its sequence is predicted to form a canonical pre-miRNA hairpin. Intrigued by this observation, the authors investigated miR858 in other plant species and found that, even though miR858 is present in all main classes of seed plants, its precursor could not be identified by de novo prediction in the majority of species. To find the elusive precursor, Wang et al. conducted RNA folding predictions of full-length transcriptome data in kiwi fruit, and identified a non-canonical hairpin with a long irregular loop that could give rise to miR858. Using transient assays, the authors proved that the long precursor can be efficiently processed into mature miR858, and its expression represses an artificial target. Furthermore, Wang et al. also found that a similarly long-looped hairpin containing the previously identified short pre-miR858a (Sharma et al. 2016) is the main source of mature miR858 in the model plant *Arabidopsis*. Overexpression of the long precursors, whose processing depends on the canonical miRNA biogenesis factor DICER-LIKE1 (DCL1), causes the repression of endogenous miR858 targets and affects flavonoid metabolism both in *Arabidopsis* and kiwi fruit (Wang et al. 2024).

A few pre-miRNAs with long hairpins have been previously reported, but they are the product of recent inverted duplications and regulate species-specific traits (Huang et al. 2017). Long pre-miR858s are different. The long stem of the hairpin is highly conserved across seed plants (Wang et al. 2024), and so are the MYB transcription factors of the R2R3 family that miR858 directly regulates (Sharma et al. 2016; Deng and Lu 2017). Nevertheless, the sequence and even the length of miR858 precursor loops varies enormously among species, ranging from ∼300 to more than 4,000 nucleotides. Interestingly, Wang et al. (2024) found different types of transposable elements in the loops of different plant taxa (Fig.).

**Figure.**
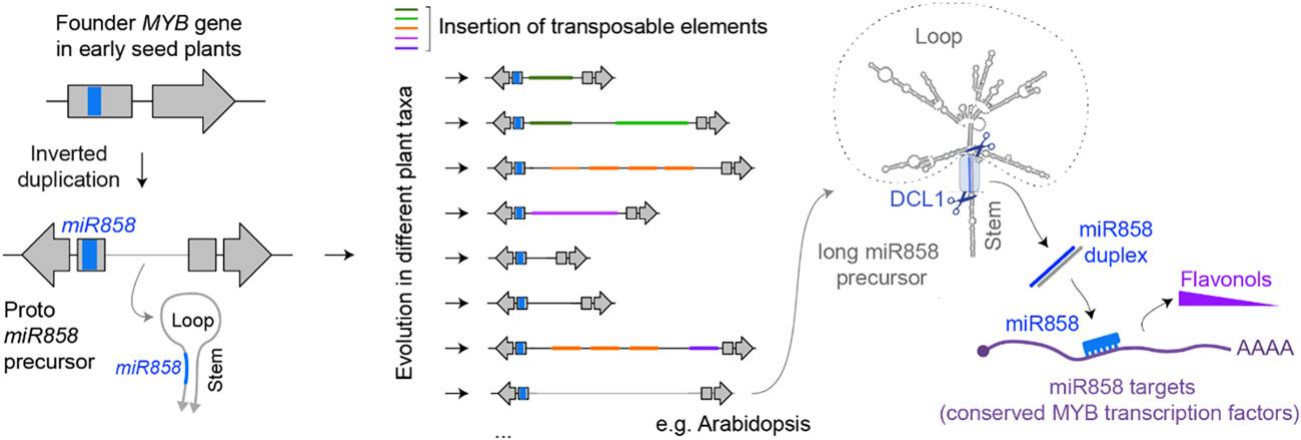
Wang et al. (2024) found that non-canonical miRNA precursors with long irregular loops, often containing transposable elements, give rise to miR858 in the main clades of seed plants. miR585 is a highly conserved miRNA that targets MYB transcription factors in control of flavonol biosynthesis. Figure credit: L. Arribas-Hernández.

It is still not clear what causes the insertion of transposable elements in pre-miR858 loops, or what the function of long and irregular loops may be. In fact, Wang et al. have shown that the conserved stem, not the long loop, is necessary for a high processing efficiency. It is also unknown whether miR858 is one of a kind, or whether other conserved miRNAs may originate from long non-canonical precursors. The increasing availability of full-transcript sequencing data from different species will surely provide answers to some of these questions. Meanwhile, it may be wise to revise the maximum allowed hairpin size in bioinformatic tools.

## Dive Curated Terms

The following phenotypic, genotypic, and functional terms are of significance to the work described in this paper:

DCL1 Gramene: AT1G01040
DCL1 Araport: AT1G01040

miR858 Gramene: AT1G71002
miR858 Araport: AT1G71002

kiwifruit Gramene: Actinidia deliciosa
kiwifruit Araport: Actinidia deliciosa
